# Supplementary material for: Therapeutic Vulnerability to ATR Inhibition in Concurrent NF1 and ATRX-Deficient/ALT-Positive High-Grade Solid Tumors
Source: Cancers (Basel). 2022 Jun 19;14(12):3015. doi: 10.3390/cancers14123015 (PMC9221513; doi:10.3390/cancers14123015)

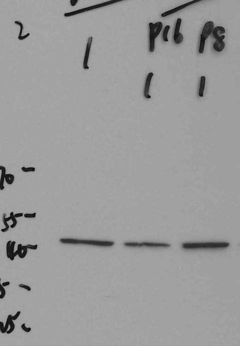
2
1
P16 P8
1 1
55
40

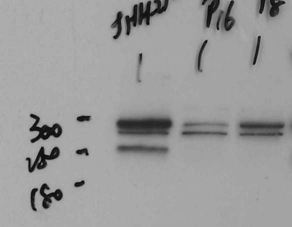

300
250
150

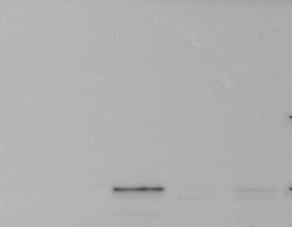

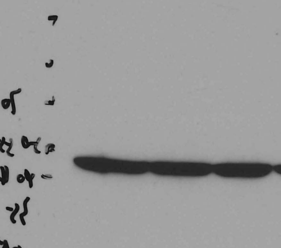

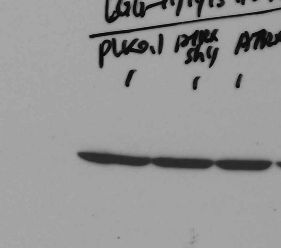
pLKO.1

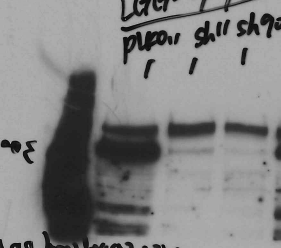
pLKO.1
sh11

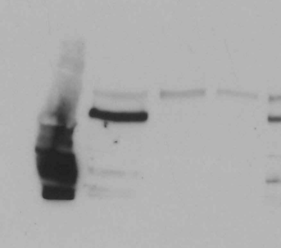

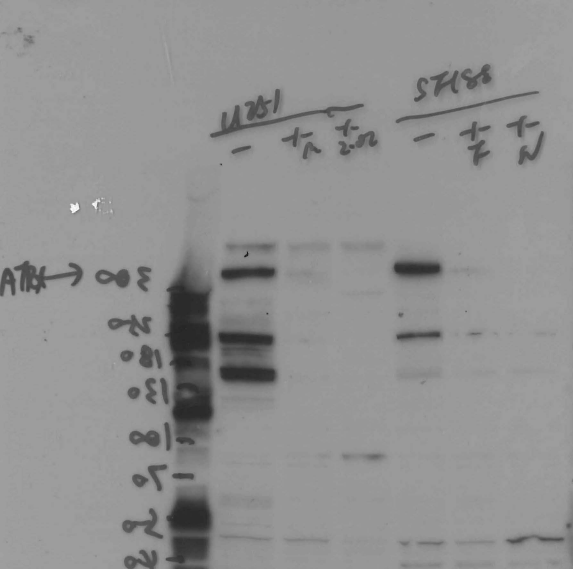
U251
SF188
ATRX

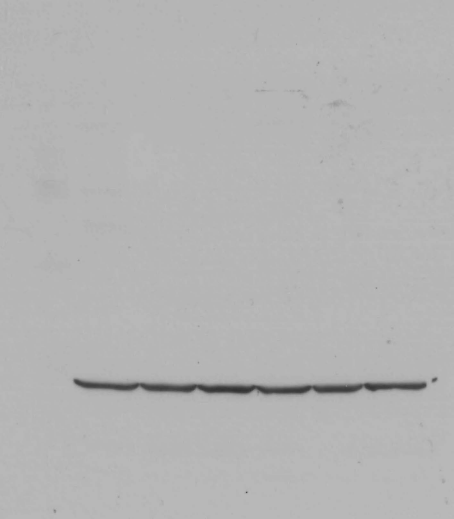

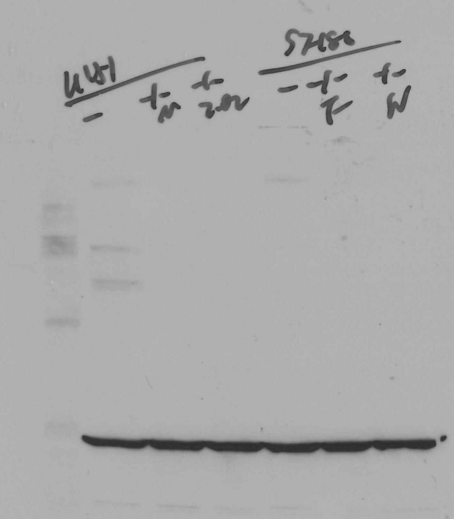

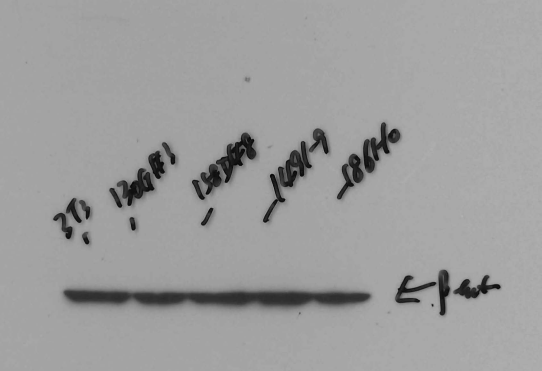
3T3

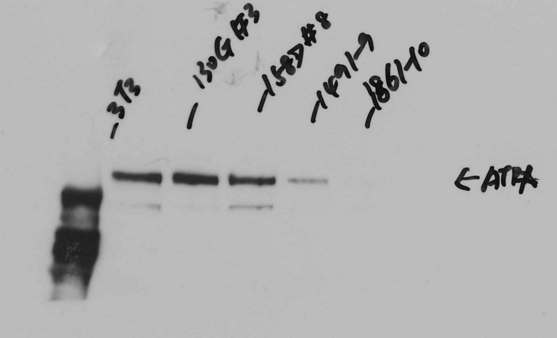
3T3
130G #3
1587 #8
1491-9
1861-10
← ATF4

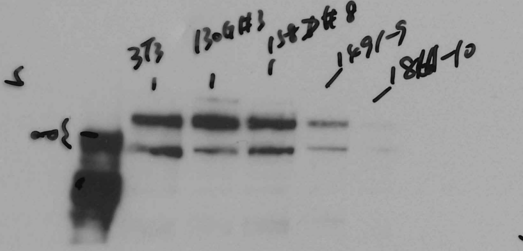
3T3
130G#3

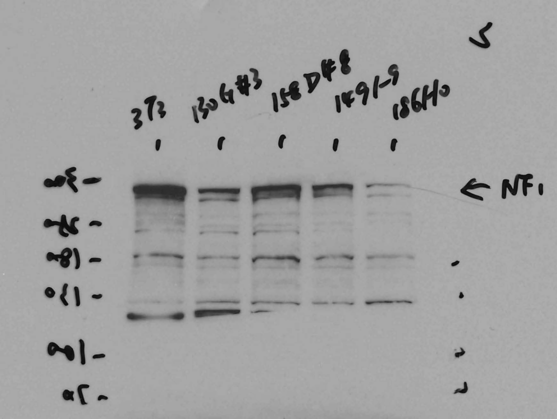
5
3T3
130G#3
158D#8
1491-9
186H0
NF1

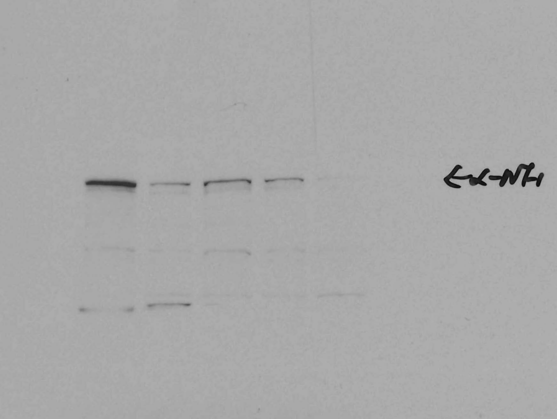
← α-NFI

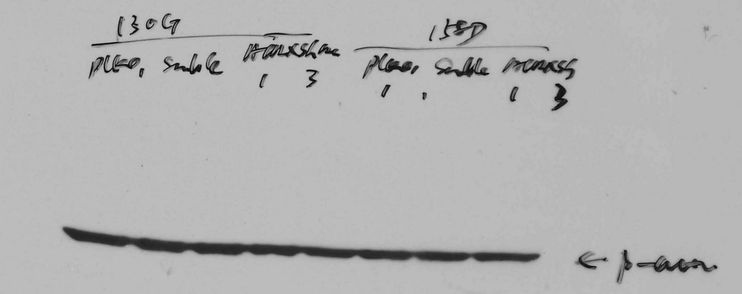

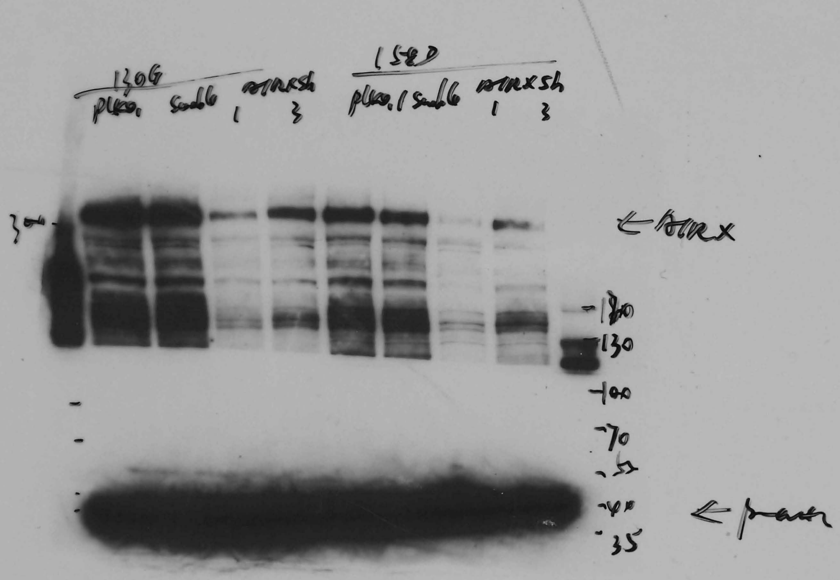
pLKO.1
1
3
180
130
100
70
40
35
ATRX

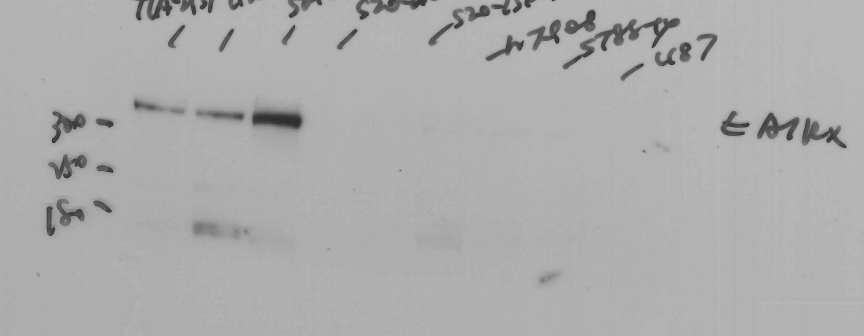
300
250
150
ST88-14
U87
← ATRX

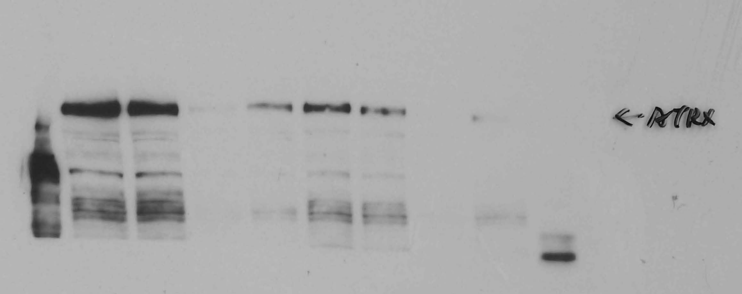

← ATRX

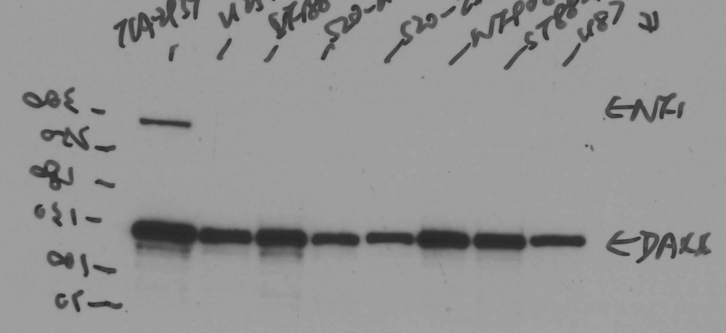
U87

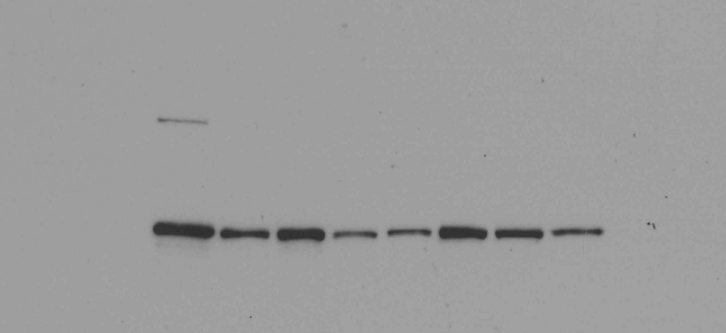

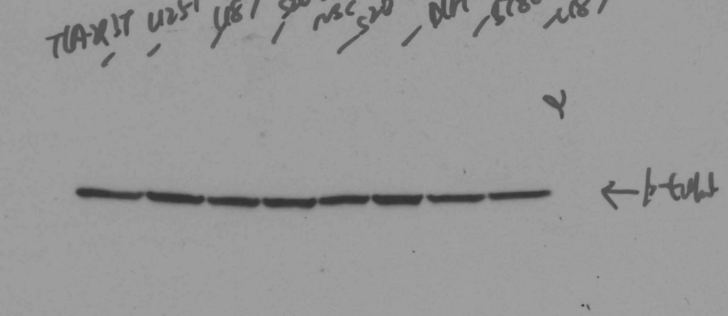

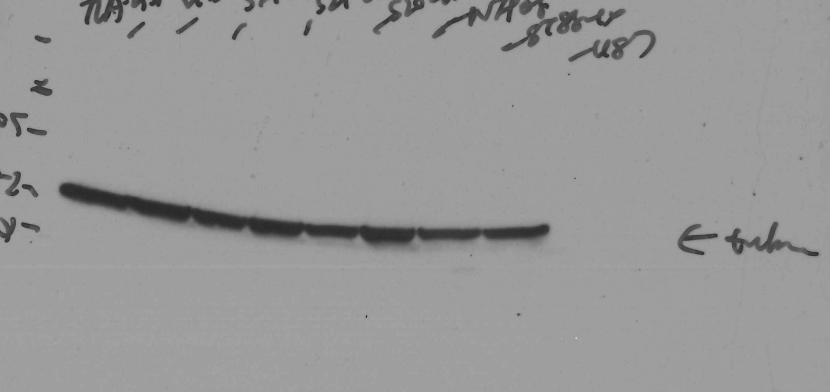
U87

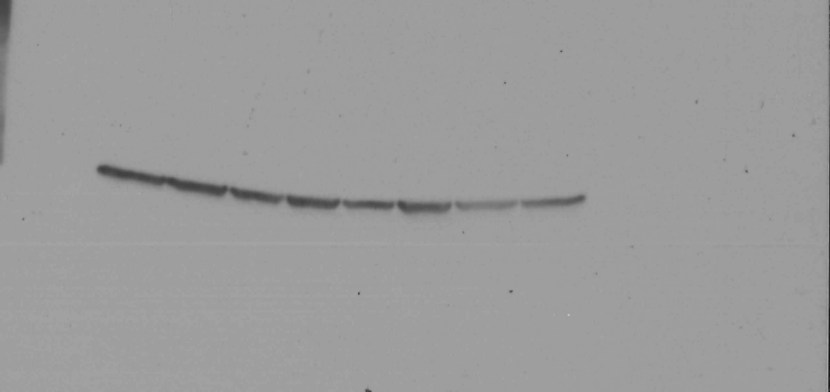

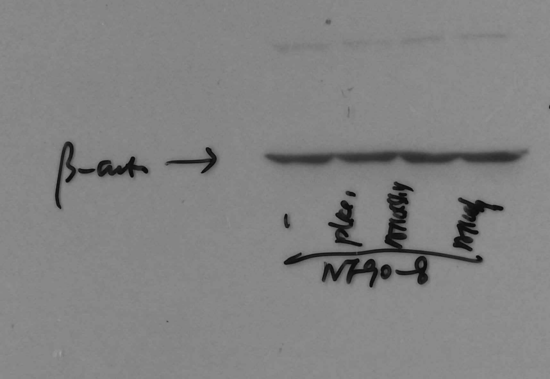
NF90-8

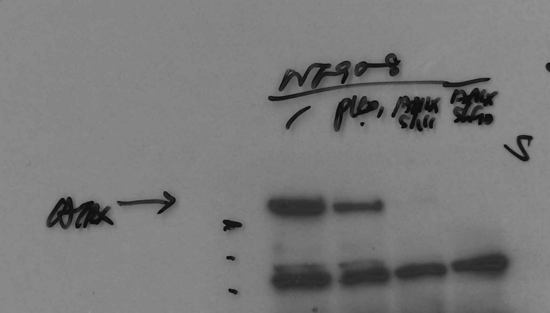

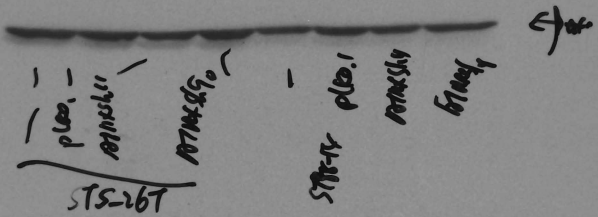
STS-26T

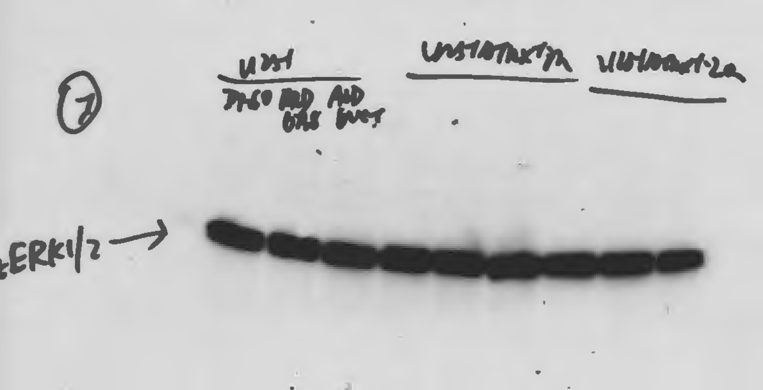

7
U251
DMSO
ERK1/2

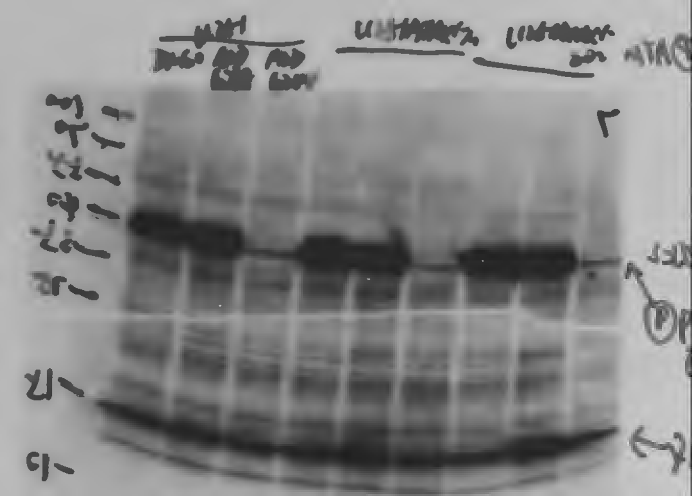

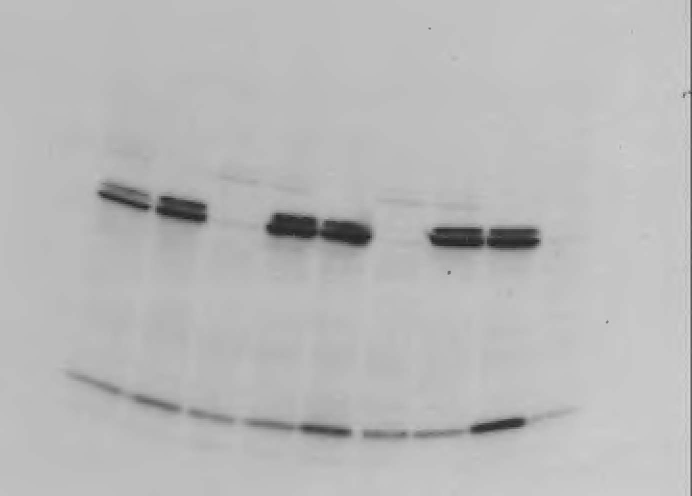

Supplement: Supplementary file 1 [file cancers-14-03015-s001.zip › cancers-1784743-File S1.pdf]
