# Supplementary material for: Therapeutic Vulnerability to ATR Inhibition in Concurrent NF1 and ATRX-Deficient/ALT-Positive High-Grade Solid Tumors
Source: Cancers (Basel). 2022 Jun 19;14(12):3015. doi: 10.3390/cancers14123015 (PMC9221513; doi:10.3390/cancers14123015)

Supplementary Figure S1. Atrx knockdown in *Nf1* +/-*Tp53* +/- murine glioma lines (130G#3 and 158D#8) did not affect cell growth. These cell lines demonstrate variable Atrx expression (A). Two lines with Atrx expression were used for knockdown experiments (B). There were no significant effects on cell growth (C).

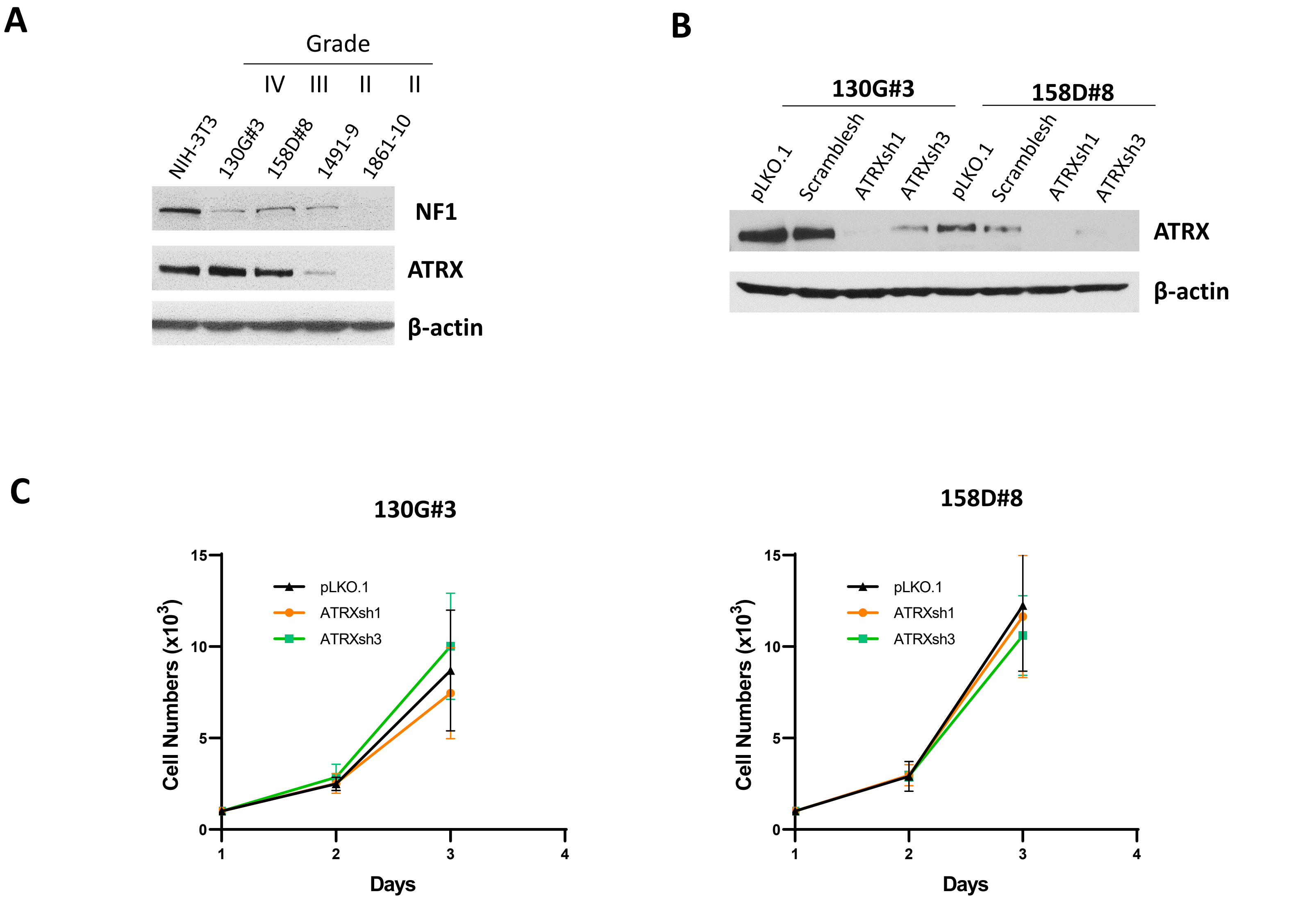

Supplementary Figure S2. ATRX knock down in human pediatric glioma cell line JHH-NF1-PA1. Short term ATRX loss resulted in upregulation of senescence markers (CDKN1A and CDKN1B), decreased *BCL2*, increased *MCL1* and *PARP1*. Empty lentiviral vector (pLKO.1) was used as control. Data was presented as mean ± s.d.(* p<0.05, ** p<0.01).

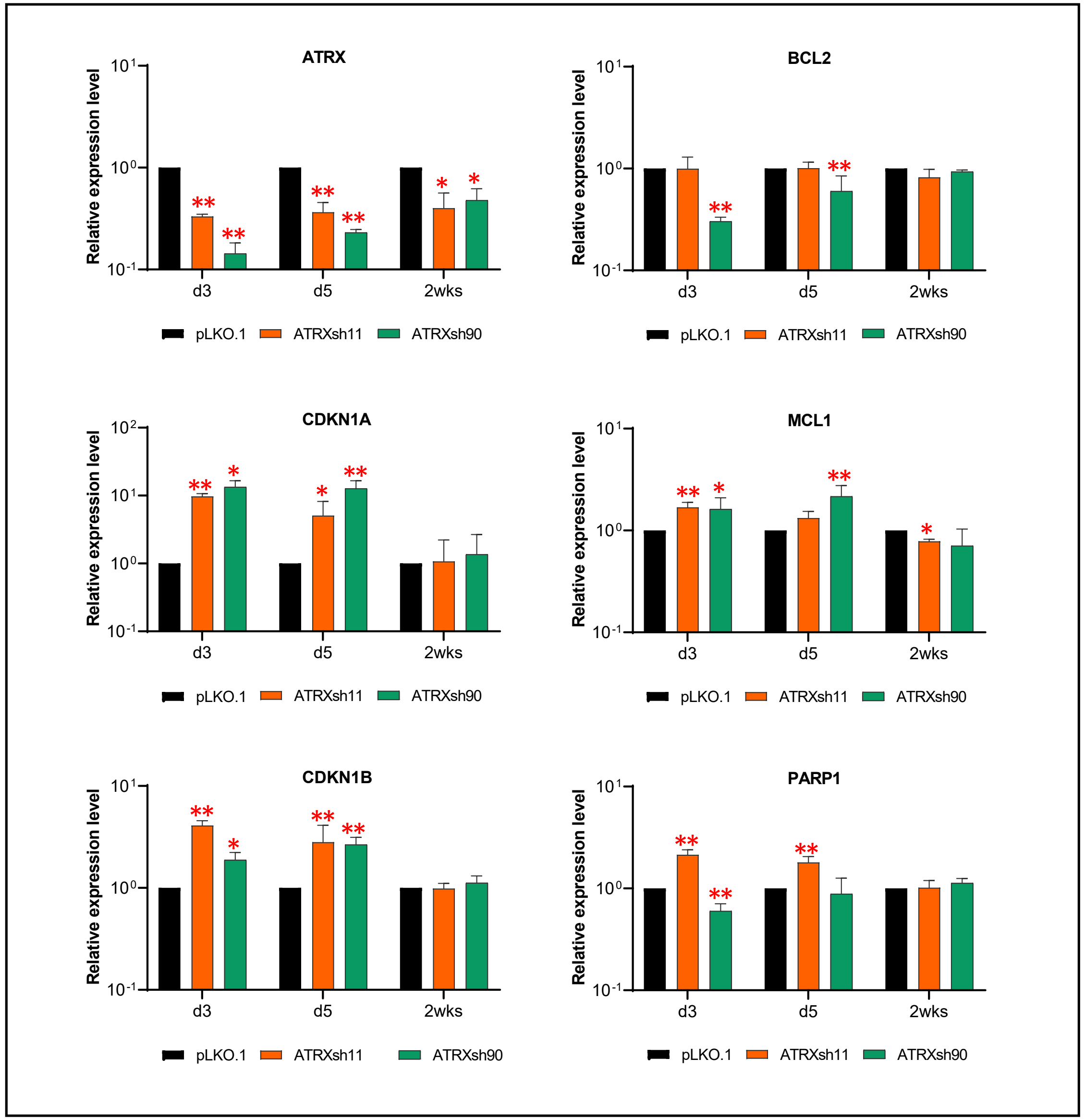

Supplementary Figure S3. NF1-deficient malignant cell lines and ATRX expression. Cell lines studied included sporadic glioma cell line U251 and SF188, NF1-patient derived cell line JHH-NF1-GBM1, NF1-patient derived sarcoma line JHH-CRC65, and MPNST cell lines NF90.8 and ST88-14. JHH-NF1-GBM1 was derived from a glioblastoma in a NF1 patient. In addition, there was complete ATRX protein loss and an *ATRX* mutation.

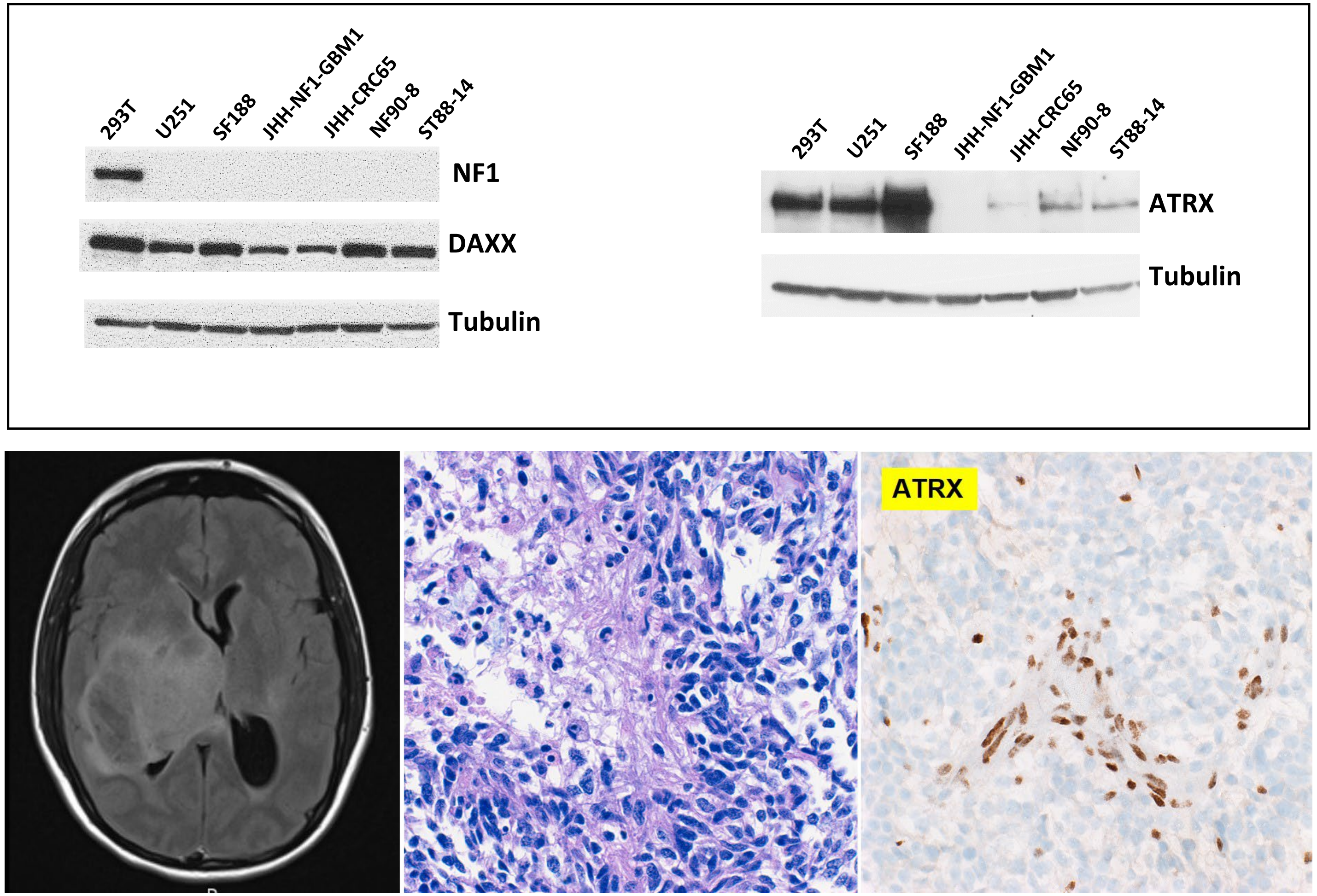

Supplementary Figure S4. ATRX knockout in NF1-deficient high-grade glioma *in vivo*. ATRX knockout resulted in less tumor burden after ATRX loss. Representative coronal sections of murine brains representing orthotopic xenografts of cell line U251 with and without ATRX knockout are presented.

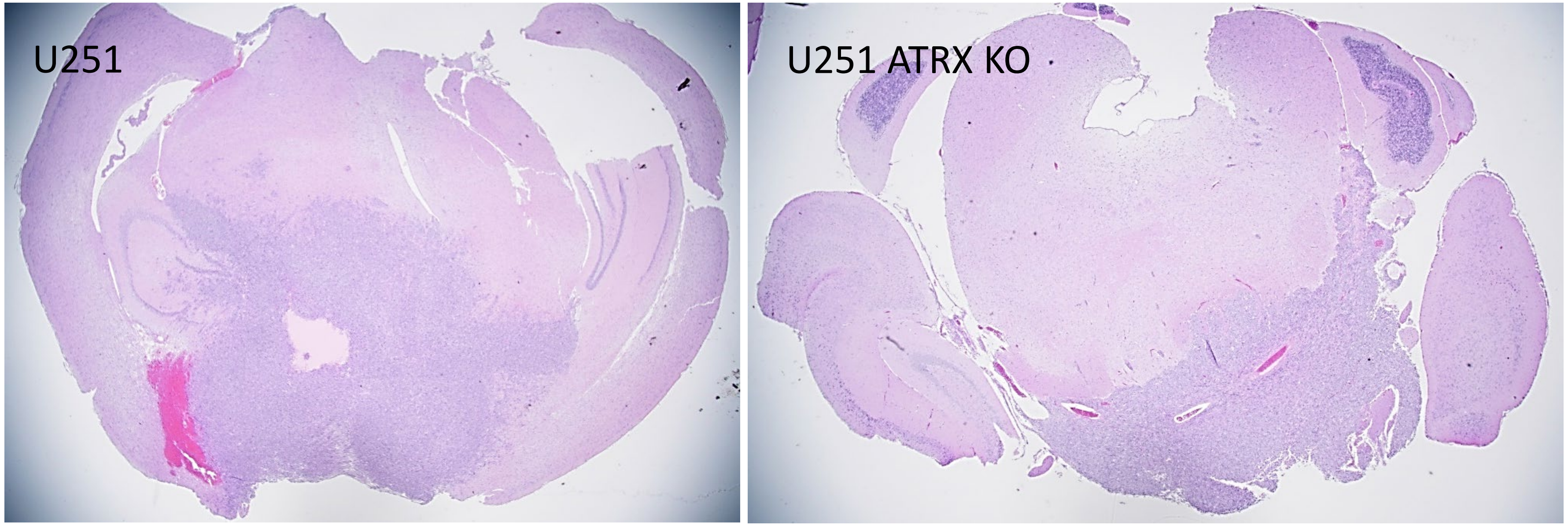

Supplementary Figure S5. ATRX loss and ALT in NF1-deficient cell lines. ATRX loss by knockout (U251) or preexisting mutation (JHH-NF1-GBM1) resulted in ALT in these two glioma cell lines as demonstrated by a c-circle assay. By contrast, NF1-sarcoma derived cell line JHH-CRC65 was ALT negative. Appropriate positive control (U2OS ALT-positive osteosarcoma cell line) and negative controls are shown at the top.

1	U251
2	U251/TERCKO#2
3	U251/TERCKO#8
4	U251
5	U251 ATRX-/-M/TERCKO#13
6	U251 ATRX$^{-/-M}$ /TERCKO#33
7	U251 ATRX$^{-/-M}$ /TERCKO#42
8	U251 ATRX$^{-/-2.02}$
9	U251 ATRX$^{-/-2.02}$ /TERCKO#30
10	U251 ATRX$^{-/-2.02}$ /TERCKO#51
11	JHH-NF1-GBM1
12	JHH-CRC65

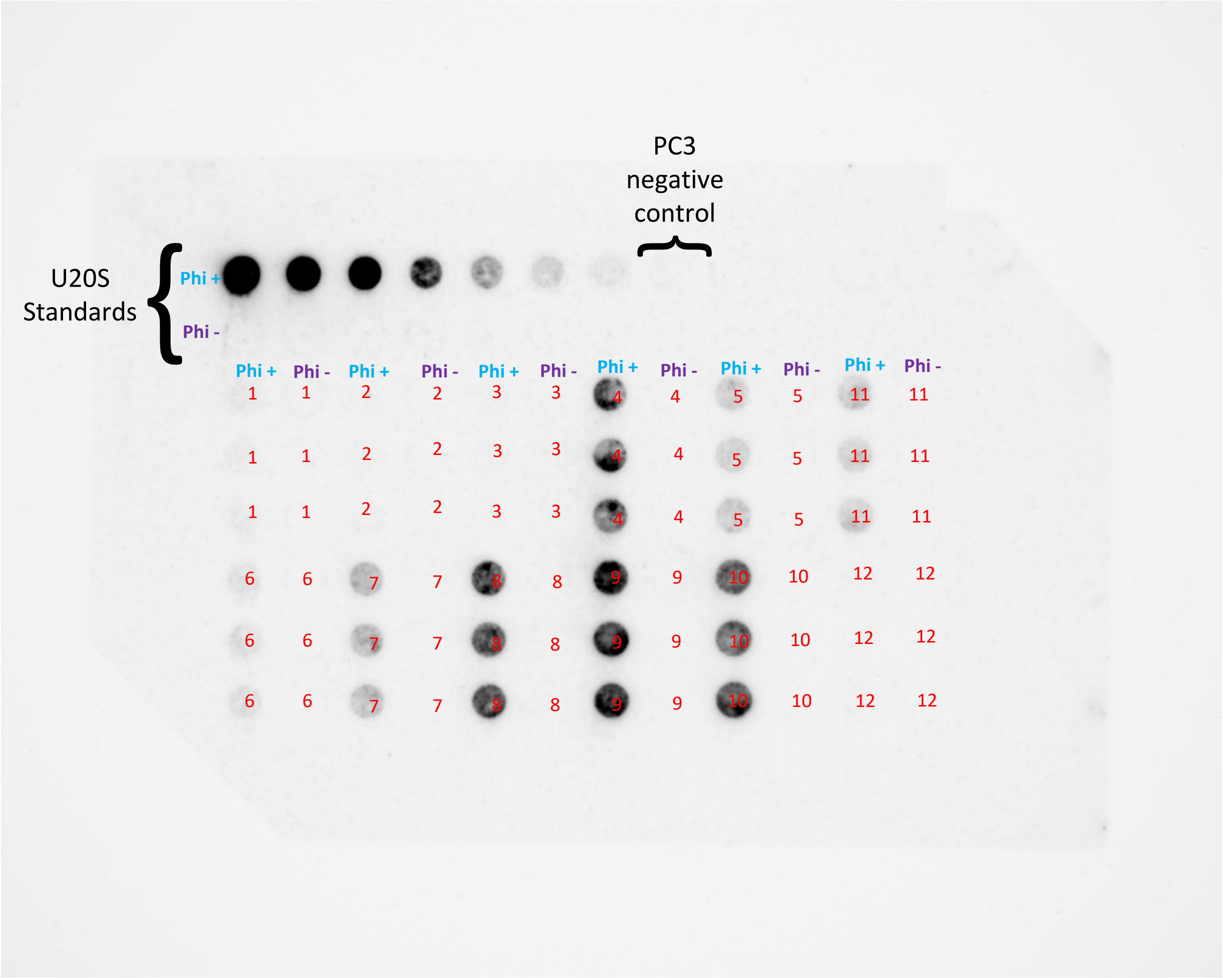

Supplementary Figure S6. ATRX knockdown in NF90-8 has no effect on cell growth. Successful ATRX knockdown in the MPNST cell line NF90-8 has no significant effect on cell growth *in vitro* (A) or *in vivo* (B). Illustrated xenografts were performed with perisciatic nerve injections.

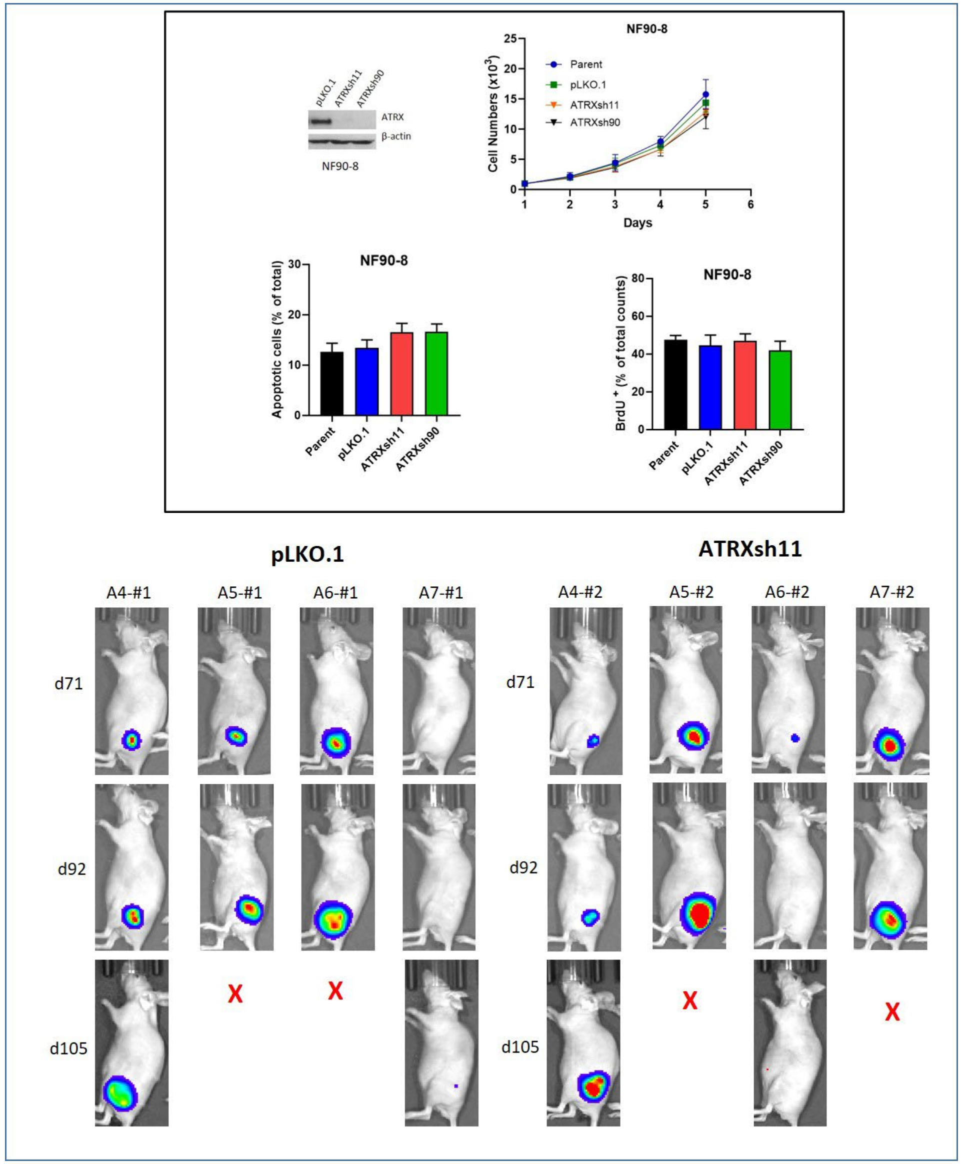

Supplementary Figure S7. ATRX knockdown in ST88-14 has no effect on cell growth. Successful ATRX knockdown in the MPNST cell line ST88-14 has no significant effect on cell growth *in vitro*.

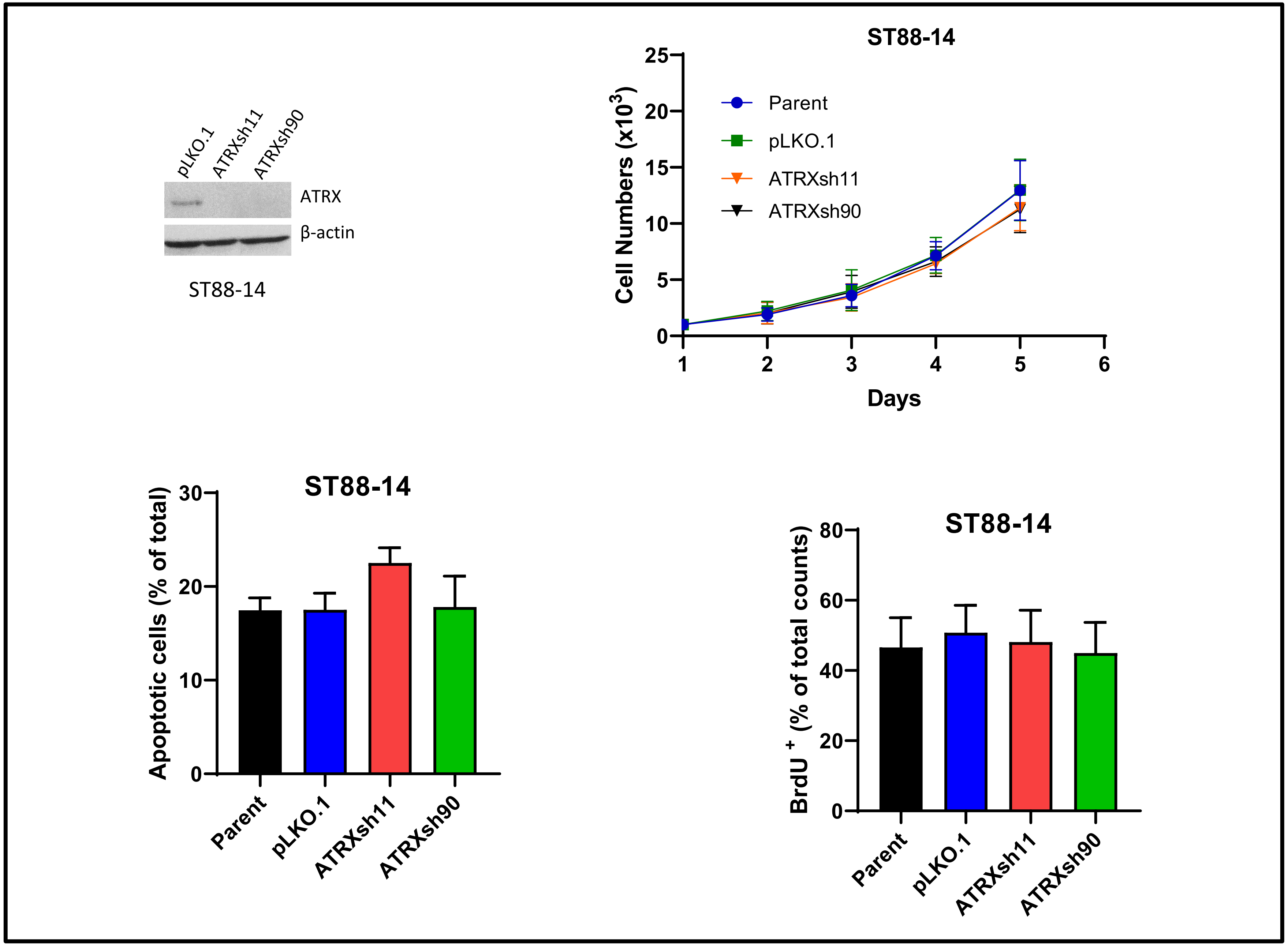

Supplementary Figure S8. ATRX knockdown in NF1 sarcoma cell line has no effect on cell growth. Successful ATRX knockdown in NF1 associated sarcoma cell line JHH-CRC65 had no significant effect on cell growth *in vitro*. Significant decreases in ATRX levels but no effect on cell growth as demonstrated by the CellTiter-Blue assay (top) were detected. There was no significant effect on apoptosis (Annexin V+) or proliferation (BrdU+ cells, bottom).

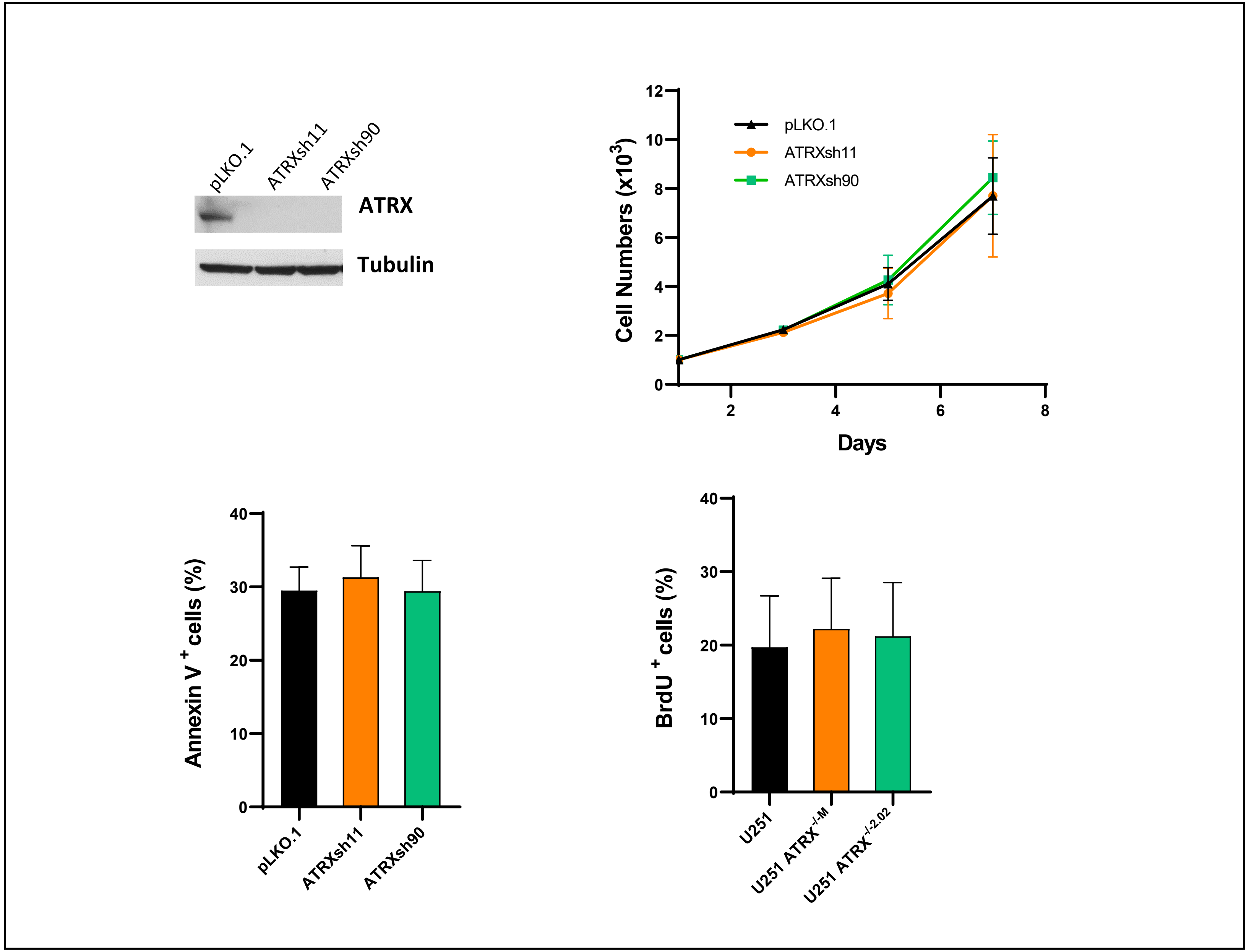

Supplementary Figure S9. ATRX knockdown leads to senescence in non-neoplastic Schwann cells. In contrast to MPNST cell lines, ATRX knockdown in a commercially available Schwann cell line resulted in senescence as demonstrated by increased mRNA levels of senescence marker CDKN1A.

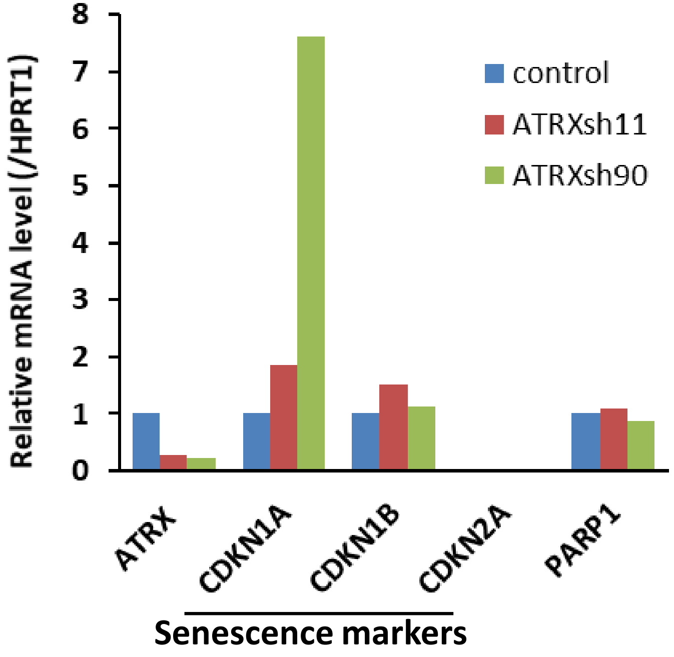

Supplementary Figure S10. ATR inhibitor sensitizes U251 cells to temozolomide. U251 cells were treated with various doses of AZD6738, temozolomide or combination for 5 days, cell survivals were normalized with vehicle control (untreated).

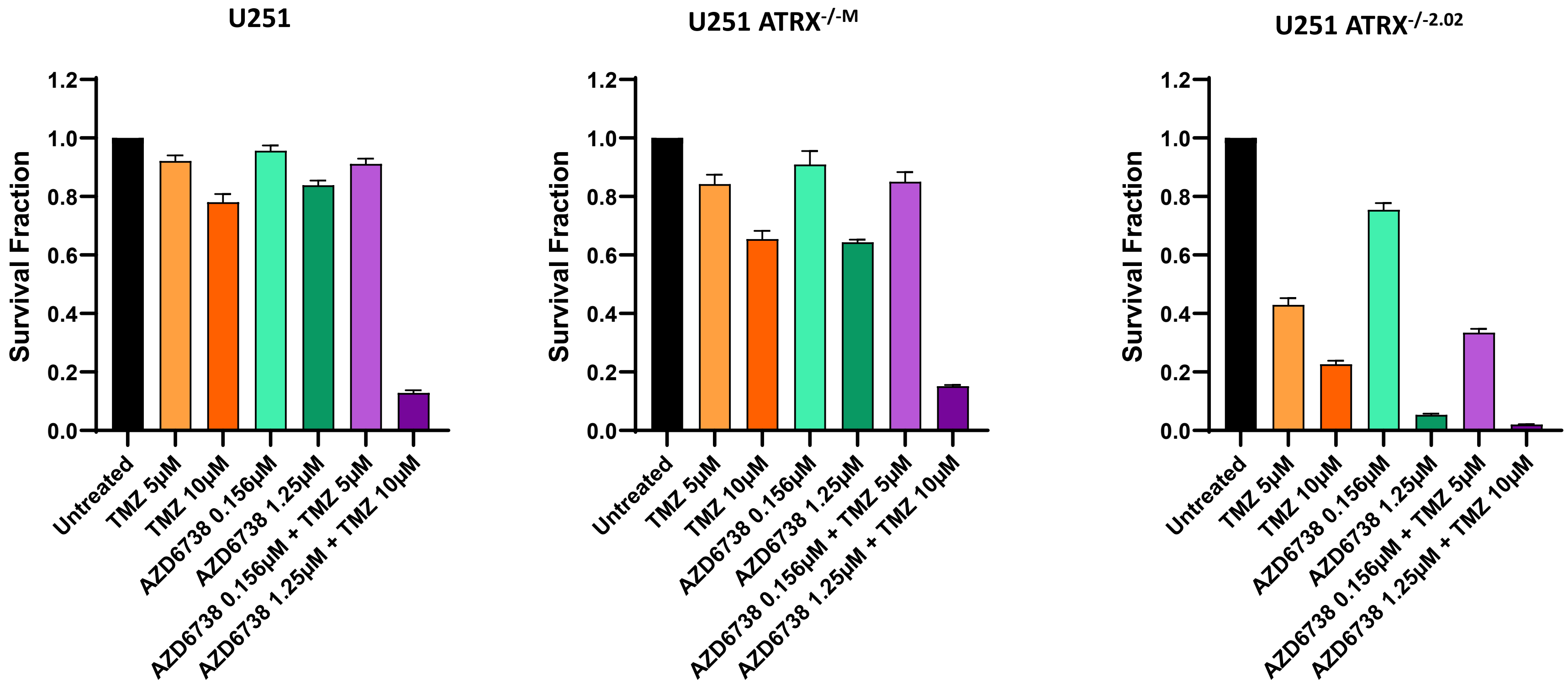

Supplementary Figure S11. ***ATRX*** **knockout sensitizes glioma cells to ATR inhibitor (AZD6738) but not MEK inhibitor (AZD6244).** MEK inhibitor AZD6244 results in decreased pERK, but not gH2AX levels (A). ATR inhibition did not sensitize U251 cells to MEK inhibition (AZD6244) (B).

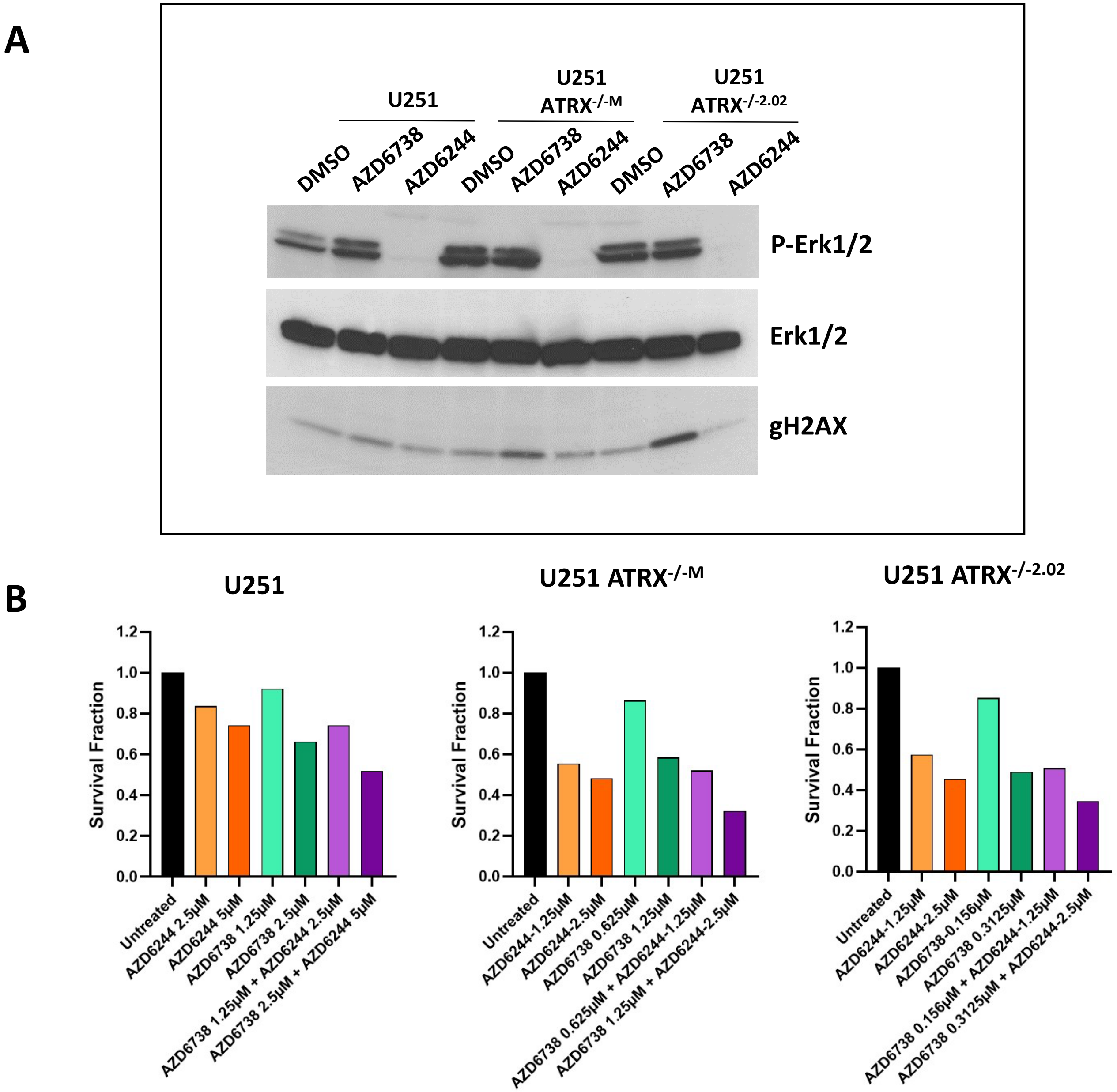

Supplementary Figure S12 . ATR inhibitor sensitizes NF90-8 cells to temozolomide. NF90-8 cells were treated with various doses of AZD6738, temozolomide or combination for 5 days. Cell survival was normalized with vehicle control (untreated).

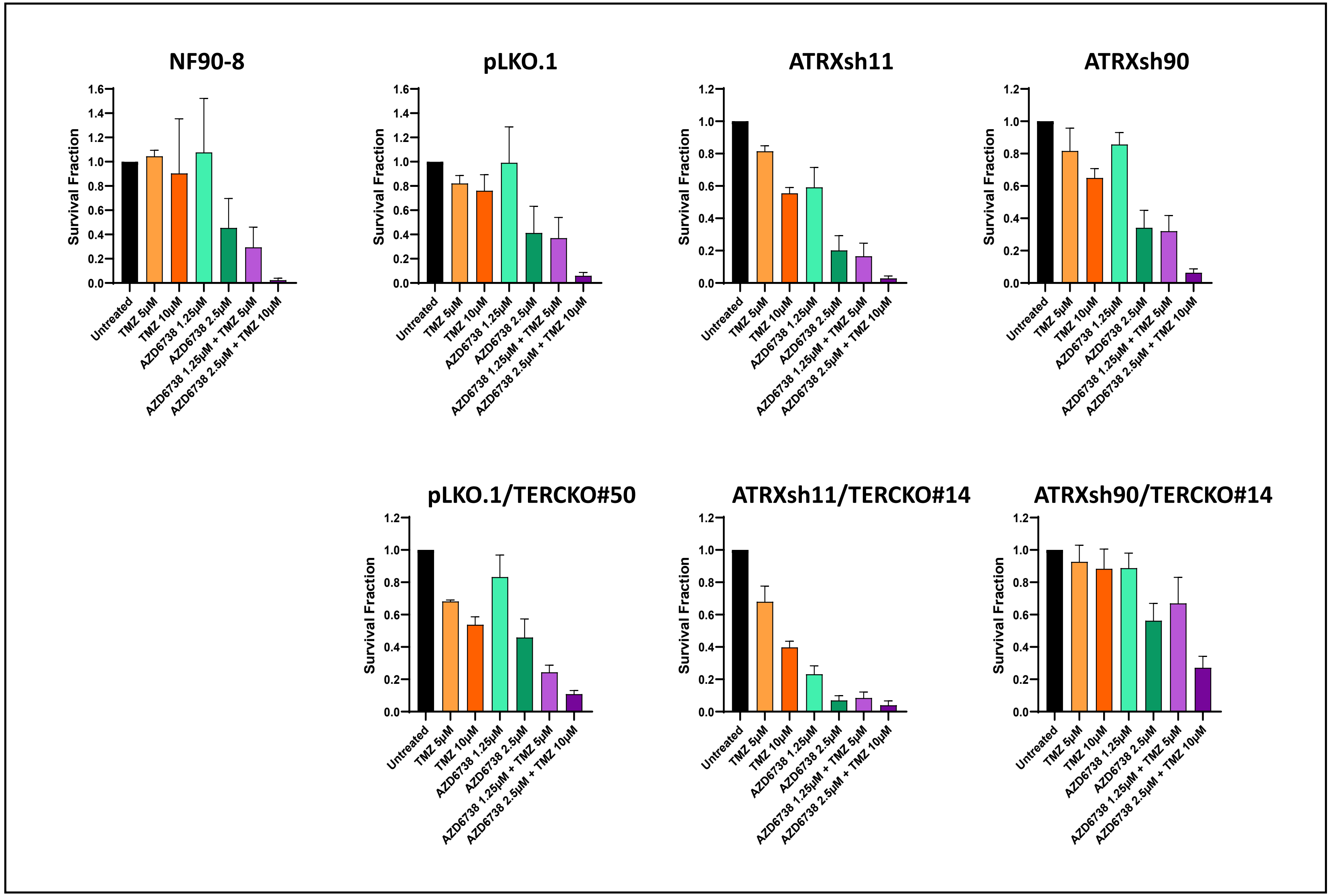

Supplementary Figure S13. *TERC* knockout in NF90-8 inhibits telomerase activity. TERC knockout was effective as demonstrated by pronounced decrease in *TERC* levels and telomerase activity.

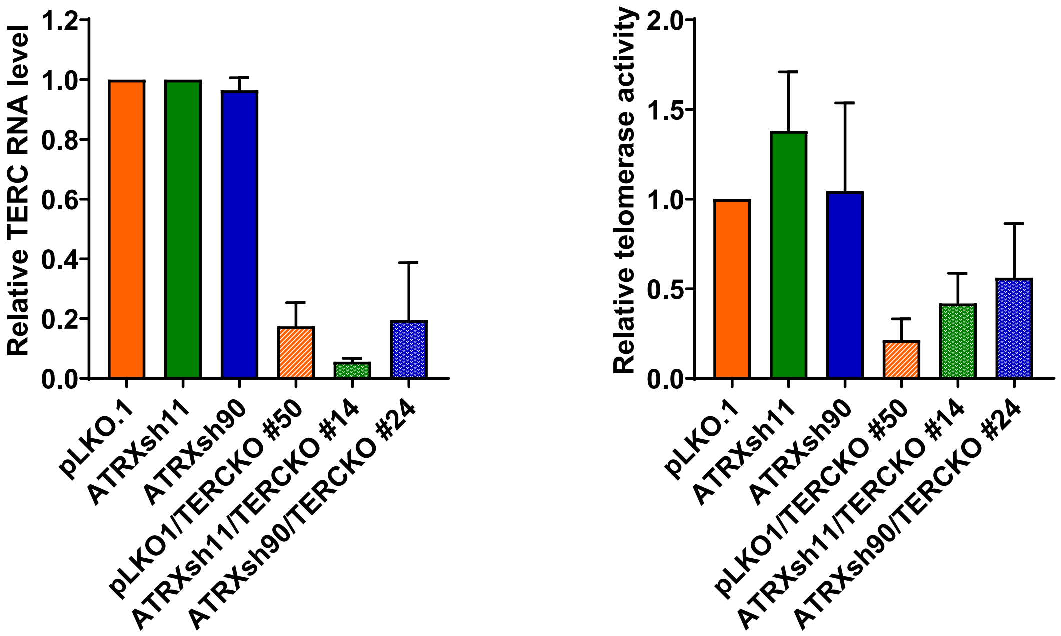

Supplementary Figure S14. ATRX knockout has no effect on cell sensitivity to ATR inhibitor (AZD6738) and temozolomide. No changes in cell growth of the NF1-derived sarcoma line JHH-CRC65 with and without ATRX knockdown after ATR inhibition or temozolomide as demonstrated by CellTiter Blue assay.

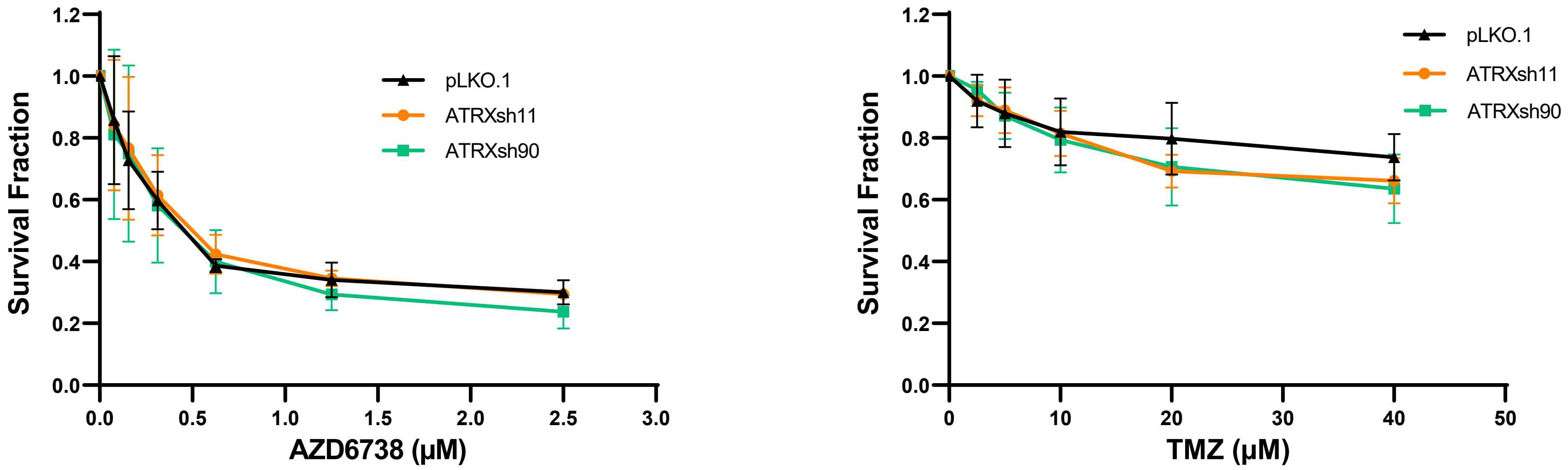

Supplement: Supplementary file 1 [file cancers-14-03015-s001.zip › cancers-1784743-supplementary.pdf]
